# MyD88 Dependent Signaling Contributes to Protective Host Defense against *Burkholderia pseudomallei*

**DOI:** 10.1371/journal.pone.0003494

**Published:** 2008-10-23

**Authors:** W. Joost Wiersinga, Catharina W. Wieland, Joris J. T. H. Roelofs, Tom van der Poll

**Affiliations:** 1 Center for Infection and Immunity Amsterdam (CINIMA), Academic Medical Center, Amsterdam, the Netherlands; 2 Center for Experimental and Molecular Medicine, Academic Medical Center, Amsterdam, the Netherlands; 3 Department of Pathology, Academic Medical Center, Amsterdam, the Netherlands; University of California Merced, United States of America

## Abstract

**Background:**

Toll-like receptors (TLRs) have a central role in the recognition of pathogens and the initiation of the innate immune response. Myeloid differentiation primary-response gene 88 (MyD88) and TIR-domain-containing adaptor protein inducing IFNβ (TRIF) are regarded as the key signaling adaptor proteins for TLRs. Melioidosis, which is endemic in SE-Asia, is a severe infection caused by the gram-negative bacterium *Burkholderia pseudomallei*. We here aimed to characterize the role of MyD88 and TRIF in host defense against melioidosis.

**Methodology and Principal Findings:**

First, we found that MyD88, but not TRIF, deficient whole blood leukocytes released less TNFα upon stimulation with *B. pseudomallei* compared to wild-type (WT) cells. Thereafter we inoculated MyD88 knock-out (KO), TRIF mutant and WT mice intranasally with *B. pseudomallei* and found that MyD88 KO, but not TRIF mutant mice demonstrated a strongly accelerated lethality, which was accompanied by significantly increased bacterial loads in lungs, liver and blood, and grossly enhanced liver damage compared to WT mice. The decreased bacterial clearance capacity of MyD88 KO mice was accompanied by a markedly reduced early pulmonary neutrophil recruitment and a diminished activation of neutrophils after infection with *B. pseudomallei*. MyD88 KO leukocytes displayed an unaltered capacity to phagocytose and kill *B. pseudomallei in vitro*.

**Conclusions:**

MyD88 dependent signaling, but not TRIF dependent signaling, contributes to a protective host response against *B. pseudomallei* at least in part by causing early neutrophil recruitment towards the primary site of infection.

## Introduction

Innate immune recognition is based on the detection of molecular structures that are unique to microorganisms [1], [2]. The Toll family of receptors (TLRs) has a central role as pattern recognition receptors (PRRs) in the initiation of cellular innate immune responses. TLRs can activate tissue-resident macrophages to produce pro-inflammatory cytokines, including TNF-α and IL-6, which coordinate local and systemic inflammatory responses [1], [3], [4]. TLR signalling depends on the selective use by different TLRs of five different adaptor molecules: myeloid differentiation primary-response gene 88 (MyD88), TIR-domain-containing adaptor protein inducing IFNβ (TRIF), MyD88-adaptor-like (MAL), TRIF-related adaptor molecule (TRAM) and sterile α- and armadillo-motifcontaining protein (SARM) [5]. MyD88 and TRIF are regarded as the main adaptor proteins. MyD88 is the key signalling adaptor for all TLRs - with the exception of TLR3 and certain TLR4 signals –, the IL-1-receptor (IL-1R) and IL-18R [5]; its main role is the activation of nuclear factor-κB (NF-κB). MyD88 is directly recruited to the TIR (Toll/IL-1R) domains in certain TLRs and acts to recruit IL-1R-associated kinase (IRAK) 4. TRIF is now known to control the TLR4-induced MyD88-independent pathway, and also to be the exclusive adaptor used by TLR3 [5]–[7]. MAL and TRAM both act as bridging adaptors, with MAL recruiting MyD88 to TLR2 and TLR4, and TRAM recruiting TRIF to TLR4 to allow for IFN regulatory factor (IRF)-3 activation [3], [5]. Finally, SARM has recently been shown to function as a negative regulator of TRIF [5], [8].

Given their central role in the recognition of microbes, TLR signalling is likely to play a crucial role in the event of sepsis: on the one hand TLR signalling is essential for the early detection of pathogens, but on the other hand can cause excessive inflammation after uncontrolled stimulation [9]–[11]. *Burkholderia pseudomallei*, a gram-negative bacterium that causes melioidosis and a recognized biological threat agent, is one of the most important causes of pneumonia-derived and community-acquired sepsis in South-East Asia and northern-Australia [12], [13]. We have recently shown that both TLR2 and TLR4 contribute to cellular responsiveness to *B. pseudomallei in vitro*, while only TLR2 impacts on the immune response of the intact host *in vivo* [14]. In the present study we sought to determine the contribution of MyD88 and TRIF in the innate immune response to *B. pseudomallei* and found that MyD88, but not TRIF, signalling plays a crucial protective role in experimental melioidosis at least in part by causing early neutrophil recruitment to the site of infection.

## Materials and Methods

### Mice

Pathogen-free 10 week old C57BL/6 wild-type (WT) mice were purchased from Harlan Sprague Dawley Inc. (Horst, the Netherlands). MyD88 knockout (KO) mice backcrossed 6 times to a C57BL/6 genetic background were generously provided by Dr. Shizuo Akira (Osaka University, Japan) [15]. Mice deficient in TRIF, generously provided by Dr. Bruce Beutler (Scripps Research Institute, La Jolla, CA), were obtained by inducing random germline mutations in C57BL/6 mice by using N-ethyl-N-nitrosourea [16]. Age and sex-matched animals were used in all experiments. The Animal Care and Use of Committee of the University of Amsterdam approved all experiments.

### In vitro stimulation

Whole blood, obtained from uninfected WT, MyD88 KO and TRIF mutant mice by bleeding from the inferior vena cava, was stimulated with heat-killed *B. pseudomallei* strain 1026b (5×10^7^ CFU/ml) or RPMI for 16 hrs as described [14], [17]. Supernatants were harvested and stored at −20°C until assayed for TNFα release.

### Experimental infection

For preparation of the inoculum, *B. pseudomallei* strain 1026b [14], [18], [19] was used and streaked from frozen aliquots into 50 ml Luria broth (Difco, Detroit, MI) for overnight incubation at 37°C in a 5% CO_2_ incubator. Thereafter, a 1 ml portion was transferred to fresh Luria broth and grown for ±5 h to midlogarithmic phase. Bacteria were harvested by centrifugation at 1500×g for 15 minutes, washed and resuspended in sterile isotonic saline at a concentration of 5×10^2^ CFUs/50 µl, as determined by plating serial 10-fold dilutions on blood agar plates. We used the inoculation dose of 5×10^2^ CFU *B. pseudomallei* to be able to compare our results with our previous studies in TLR2 and TLR4 KO mice in which we used the exact same dose [14]. Pneumonia was induced by intranasal inoculation of 50 µl (5×10^2^ CFU) bacterial suspension. For this procedure mice were lightly anesthetized by inhalation of isofluorane (Upjohn, Ede, The Netherlands).

### Determination of bacterial outgrowth

At designated time points after infection, mice were anesthetized with Hypnorm® (Janssen Pharmaceutica, Beerse, Belgium: active ingredients fentanyl citrate and fluanisone) and midazolam (Roche, Mijdrecht, The Netherlands) and sacrificed by bleeding from the inferior vena cava. The lungs and liver were harvested and homogenized at 4°C in 4 volumes of sterile saline using a tissue homogenizer (Biospec Products, Bartlesville, OK). CFUs were determined from serial dilutions of organ homogenates and blood, plated on blood agar plates and incubated at 37°C at 5% CO_2_ for 16 h before colonies were counted.

### Assays

Lung homogenates were prepared as described earlier [14], [20], [21]. Mouse TNF-α, IFN-γ, monocyte chemoattractant protein (MCP)-1, IL-6 and IL-10 were measured by cytometric bead array (CBA) multiplex assay (BDBiosciences, San Jose, CA) in accordance with the manufacturer's recommendations. Myeloperoxidase (MPO; HyCult Biotechnology, Uden, the Netherlands), lipopolysaccharide-induced CXC chemokine (LIX; R&D Systems, Minneapolis, MN), KC and macrophage-inflammatory protein-(MIP)-2 (both RnD systems, Minneapolis, MN) were measured using commercially available ELISA's or antibody pairs. Aspartate aminotransferase (ASAT) and alanine aminotransferase (ALAT) were determined with commercially available kits (Sigma-Aldrich), using a Hitachi analyzer (Roche) according to the manufacturer's instructions.

### Pathology

Lungs and liver for histology were prepared and analyzed as described earlier [14], [20], [21]. To score lung inflammation and damage, the entire lung surface was analyzed with respect to the following parameters: surface with pneumonia, necrosis/abscess formation, interstitial inflammation, endothelialitis, bronchitis, edema, thrombus formation and pleuritis. Each parameter was graded on a scale of 0 to 4, with 0: absent, 1: mild, 2: moderate, 3: severe, 4: very severe. The total “lung inflammation score” was expressed as the sum of the scores for each parameter, the maximum being 32. Liver sections were scored on inflammation, necrosis/abscess formation and thrombus formation using the scale given above.

### Flow cytometry

Lung cell suspensions were obtained by passing the lungs trough a 40-µm cell strainer (BD, Franklin Lakes, NJ) as described previously [22], [23]. Erytrocytes were lysed with ice-cold isotonic NH_4_Cl solution (155 mM NH_4_Cl, 10 mM KHCO_3_, 0.1 mM EDTA, pH 7.4); the remaining cells were washed twice with RPMI 1640 (Bio Whiitaker, Verviers, Belgium), and counted by suing a hemocytometer. The percentages of macrophages, neutrophils and lymphocytes were determined using a FACSCalibur (BD, San Jose, CA). Cells were brought to a concentration of 1×10^7^ cells/ml in FACs buffer (PBS supplemented with 0.5% PBS, 0.01% NaN_3_ and 0.35 mM EDTA). Immunostaining for cell surface molecules was performed for 30 minutes at 4°C using directly labeled antibodies (abs) against GR-1 (GR-1 FITC, BDPharmingen, San Diego, California), CD11b (CD11b-phycoerythrin, BDPharmingen) and a biotin labeled antibody against F4/80 (Serotec, Oxford, United Kingdom) in combination with streptavidin allophycocyanine. All abs were used in concentrations recommended by the manufacturer (BD Pharmingen, San Diego, CA). After staining, cells were fixed in 2% paraformaldehyde. Neutrophils were counted using the scatter pattern and GR-1 high gate, monocytes in the sidescatter low and F4/80 postive gate and macrophages in the sidescatter high and F4/80 positive gate.

### Phagocytosis and bacterial killing assays

Phagocytosis was evaluated in essence as described before [24], [25]. Concentrated *B. pseudomallei* preparation was treated for 90 minutes at 38°C with 100 µg/ml mitomycine-C (Sigma-Aldrich, Zwijndrecht, the Netherlands) to prepare alive but growth-arrested bacteria. To determine the neutrophil phagocytosis capacity, 50 µl of whole blood was incubated with carboxyfluorescein-diacetate-succinimidyl-ester (CFSE dye, Invitrogen, Breda, the Netherlands) labeled, growth-arrested bacteria (1×10^7^ CFU/ml) and incubated for 10, 60 or 120 minutes at 37°C. Cells were suspended in Quenching solution, incubated in FACS lysis/fix solution (BD, Mountain View, CA) and neutrophils were labeled using anti-Gr-1-PE (BD Pharmingen, San Diego, CA). Cells were washed with ice-cold FACS buffer after which the degree of phagocytosis of neutrophils was determined using FACSCalibur (Becton Dickinson;. 1×10^4^ neutrophils were analysed per sample). The phagocytosis index of each samples was calculated as follows: (mean fluorescence×% positive cells at 37°C) minus (mean fluorescence×% positive cells at 4°C). Bacterial killing was determined as described [26]. In brief, peritoneal macrophages were harvested from WT and MyD88 KO mice and plated as described above (n = 5 per strain). *B. pseudomallei* was added at a multiplicity of infection (MOI) of 50 and spun onto cells at 2000 rpm for 5 minutes, after which plates were placed at 37°C for 10 minutes. Each well was then washed 5 times with ice-cold PBS to remove extracellular bacteria. To determine bacterial uptake after 10 minutes, wells were lysed with sterile dH_2_O and designated as t = 0. RPMI was added to remaining wells and plates were placed at 37°C for 10 or 60 minutes after which cells were again washed 5 times with ice-cold PBS and lysed with dH_2_O. Cell-lysates were plated on blood agar plates and bacterial counts were enumerated after 16 h. Bacterial killing was expressed as the percentage of killed bacteria in relation to t = 0.

### Statistical analysis

All data are expressed as mean±SEM. Difference between groups were analyzed with Mann-Whitney *U* test or Kruskal-Wallis analysis with Dunn post hoc test where appropriate. For survival analyses, Kaplan-Meier anlyses followed by log-rank test was performed. These analyses were performed using GraphPad Prism version 4 (GraphPad Software, San Diego, CA). Values of *p*<0.05 were considered statistically significant.

## Results

### MyD88 but not TRIF is required for cellular responsiveness to B. pseudomallei in vitro

**Figure 1:**
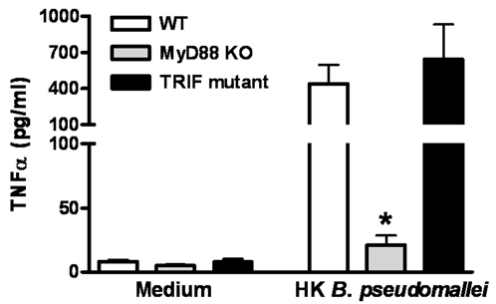
MyD88, but not TRIF, is required for cellular responsiveness to *B. pseudomallei in vitro*. Whole blood of wild-type (WT; white bars), MyD88 knock-out (KO; grey bars) and TRIF mutant (black bars) mice were incubated with RPMI 1640 medium or heat-killed (HK) *B. pseudomallei* (5×10^7^ CFU)/ml) for 16 hours before TNFα production was measured in the supernatant. Data are means±SEM (n = 5–6/strain); * *P*<0.05.

To obtain a first insight into the function of the TLR adaptor molecules MyD88 and TRIF in melioidosis, we tested the requirement of MyD88 and TRIF signaling upon first encounter between *B. pseudomallei* and the host. Therefore, we tested the capacity of whole blood harvested from WT, MyD88 KO and TRIF mutant mice to release TNFα upon stimulation with *B. pseudomallei*. MyD88 deficient leukocytes, but not TRIF deficient leukocytes, released less TNFα than WT leukocytes upon stimulation with heat-killed *B. pseudomallei in vitro* **(** **Fig. 1** **)**, suggesting that MyD88, and not TRIF, contributes to cellular responsiveness towards *B. pseudomallei in vitro*.

### MyD88 KO, but not TRIF mutant, mice show an accelerated mortality during experimental melioidosis

**Figure 2:**
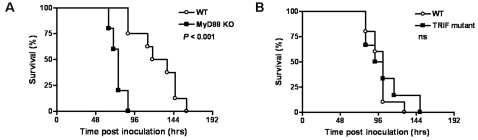
MyD88 KO, but not TRIF KO, mice show an accelerated mortality during experimental melioidosis. Survival of wild-type (WT, open rounds) and MyD88 KO mice (black squares) (A) or TRIF mutant (black squares) mice (B) intranasally infected with 5×10^2^ CFU *B. pseudomallei*. Mortality was assessed twice daily for one week. n = 8–10 per group; ns denotes not significant; *P* value indicates the difference between MyD88 KO and WT mice.

We next investigated the involvement of MyD88 and TRIF in the host response to *B. pseudomallei* infection *in vivo*. As a first experiment WT, MyD88 KO and TRIF mutant mice were intranasally infected with a lethal dose of *B. pseudomallei* and followed for one week. MyD88 deficiency had a markedly negative influence on survival: whereas all WT mice were dead after 160 hours (median survival time 118 hours), all MyD88 KO mice died within 88 hours (median survival time 76 hours; *P*<0.001 for the difference between groups; **Fig. 2A**). TRIF deficiency on the other hand did not impact on survival during experimental melioidosis **(** **Fig. 2B** **)**.

### MyD88 but not TRIF contributes to bacterial clearance of B. pseudomallei in vivo

**Figure 3:**
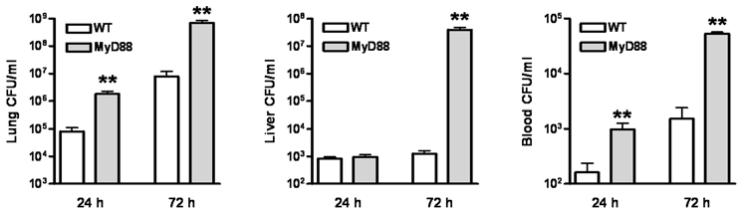
MyD88 KO mice show increased bacterial outgrowth during experimental melioidosis. WT and MyD88 KO mice were intranasally infected with *B. pseudomallei* (5×10^2^ CFU). Bacterial loads were measured 24 h and 72 h after inoculation in lungs (A), liver (B) and blood (C). Data are mean±SEM (n = 6–7 per group at each time point). ** *P*<0.01.

**Figure 4:**
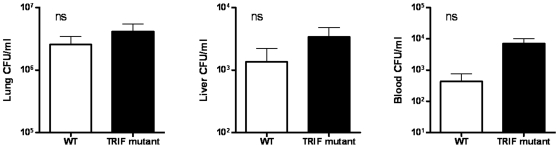
TRIF deficiency does not impact on bacterial outgrowth during experimental melioidosis. WT and TRIF mutant mice were intranasally infected with *B. pseudomallei* (5×10^2^ CFU). Bacterial loads were measured 72 h after inoculation in lungs (A), liver (B) and blood (C). Data are mean±SEM (n = 8 per group at each time point); ns denotes not significant.

To obtain insight into the mechanisms underlying the accelerated mortality of MyD88 KO mice during experimental melioidosis, we infected WT and MyD88 KO mice with *B. pseudomallei* and sacrificed them after 24 (i.e. just before the first symptoms of illness occurred) and 72 hours (i.e. before the first deaths occurred) to determine bacterial loads in lungs (the primary site of the infection), liver and blood (to evaluate dissemination to distant body sites; **Fig. 3**). Relative to WT mice, MyD88 KO mice displayed strongly increased pulmonary and systemic bacterial loads at 24 and 72 hours after infection, as well as in their livers at 72 hours **(** **Fig. 3** **)**. Conversely, similar bacterial loads in the lungs, liver and blood of WT and TRIF mutant mice were observed 72 hours after infection with *B. pseudomallei* **(** **Fig. 4** **)**.

### Role of MyD88 and TRIF in B. pseudomallei induced pulmonary inflammation

**Figure 5:**
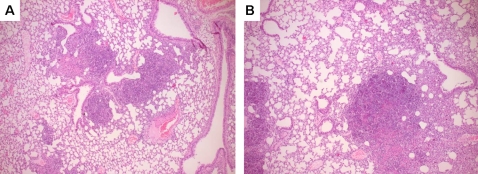
Pulmonary inflammation in mice infected with *B. pseudomallei*. Representative histopathology slides of lungs of WT (A) and MyD88 KO (B) mice infected with 5×10^2^ CFU *B. pseudomallei* at 72 h after infection showing significant infilates, edema and peribronchial inflammation. The preparations were stained with haematoxylin and eosin. Original magnification, ×50.

The success of combating infection strongly depends on the generation of an effective inflammatory response at the primary site of infection [11], [27]. Therefore, to further investigate the role of MyD88 and TRIF in the inflammatory response towards infection with *B. pseudomallei*, we first measured cytokine levels in whole lung homogenates harvested from WT, MyD88 KO and TRIF mutant mice after intranasal infection with *B. pseudomallei* **(** **Table 1** **)**. 24 h after infection, pulmonary TNFα, MCP-1 and IL-10 were lower in MyD88 KO mice relative to WT mice **(** **Table 1** **)**. At 72 h after infection TNFα levels remained lower in MyD88 KO mice, while MCP-1 and IL-6 levels tended to be higher in these animals **(** **Table 1** **)**. In addition, histological samples of lungs obtained 24 and 72 hours after infection were semi-quantitatively scored on the extent of inflammation. Pulmonary inflammation was characterized by significant inflammation, pleuritis, peribronchial inflammation, oedema and endothelialitis in both WT and MyD88 KO mice **(** **Fig. 5** **)**. MyD88 KO mice tended to have more lung injury compared to WT mice, however lung inflammation scores were not significantly different at 72 hours after infection between both mice strains (data not shown). Pulmonary cytokine levels and lung inflammation scores were similar in TRIF mutant and WT mice at all time points (data not shown).

**Table 1:** Role of MyD88 in *B. pseudomallei* induced pulmonary cytokine levels.

pg/ml	WT	MyD88 KO	WT	MyD88 KO
	t = 24		t = 72	
**TNF-α**	50±18	10±2*	1048±266	314±33**
**IFN-γ**	BD	BD	11±3	BD
**MCP-1**	993±255	245±46*	6354±1379	9950±32
**IL-6**	487±122	85±15	1418±869	2990±456
**IL-10**	120±48	28±11*	278±53	68±16

Groups of 5–7 mice were intranasally inoculated with 5×10^2^ CFU *B. pseudomallei*. After 24 and 72 h mice were sacrificed, lungs were removed, homogenized and cytokines were measured. Data are expressed as mean±SEM; BD: below detection; * *P*<0.05; ** *P*<0.01 versus WT controls.

### Enhanced liver damage in MyD88 KO mice

**Figure 6:**
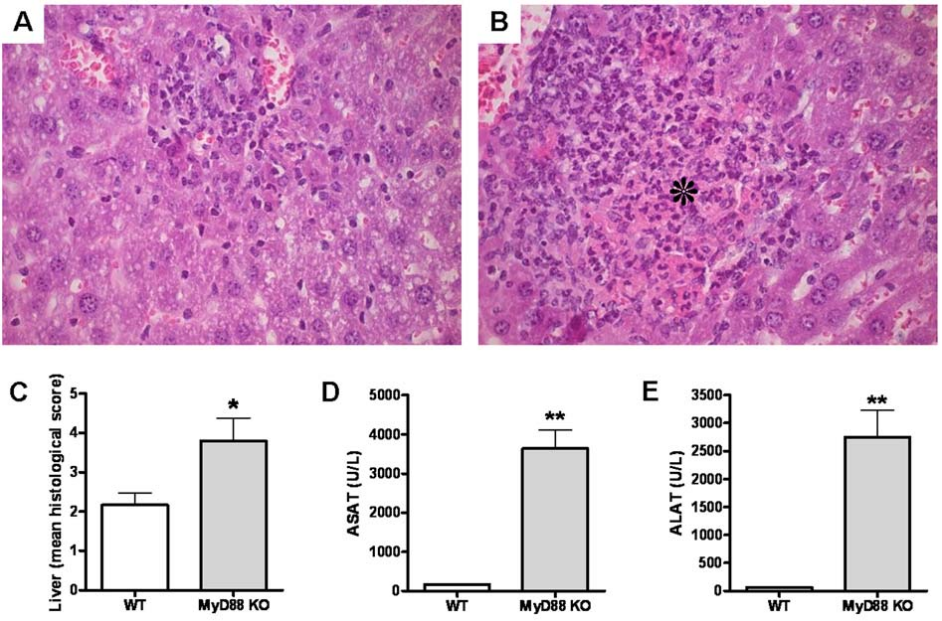
Enhanced liver damage in MyD88 KO mice. Representative hematoxylin- and eosin-stained liver histology slides for WT (A) and MyD88 KO (B) mice at 72 h after inoculation with 5×10^2^ CFU *B. pseudomallei* show more inflammation and foci of necrosis (as indicated by the asterisk) in the livers derived from MyD88 KO animals compared to WT controls (original magnification, ×400) corresponding with higher pathology scores (see Methods section) (C). At 72 h after inoculation MyD88 KO mice also showed enhanced hepatic injury, as reflected by the plasma concentrations of aspartate aminotransferase (ASAT) (D) and alanine aminotransferase (ALAT) (E). Data are mean±SEM (n = 5–6 per group); U/L, units per liter; * *P*<0.05; ** *P*<0.01.

After the lung, the liver is one of the most commonly affected organs in melioidosis [12], [28]. Considering this and the observed increased bacterial loads in the liver, we examined the influence of MyD88 deficiency on liver damage 72 h post infection. Upon histopathological examination, both WT and MyD88 KO mice showed mild inflammation of liver tissue as characterized by the influx of leukocytes into the hepatic parenchyma **(** **Fig. 6A–B** **)**. In contrast to WT mice, MyD88 KO mice showed foci of liver necrosis and the formation of small abscesses **(** **Fig. 6B** **)**. In line, the extent of hepatic inflammation as quantified according to the scoring system described in *Materials and Methods* section was significantly increased in MyD88 KO mice compared to controls **(** **Fig. 6C** **)**. The increased hepatocellular injury in MyD88 KO mice after infection with *B. pseudomallei* was further underscored by clinical chemistry: MyD88 KO mice displayed strikingly higher plasma levels of ASAT and ALAT compared to WT mice **(** **Fig. 6D–E** **)**.

### No role of MyD88 in phagocytosis of B. pseudomallei

**Figure 7:**
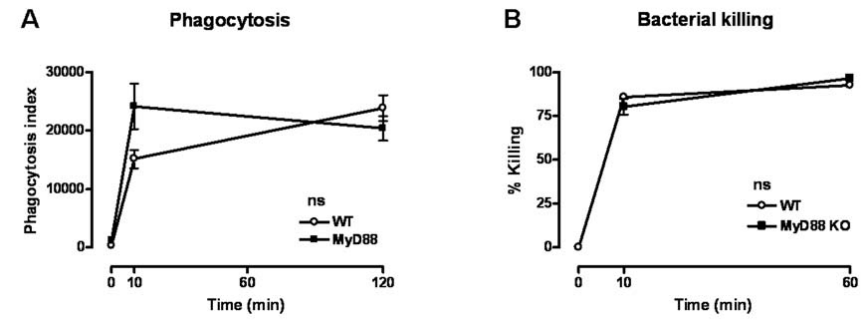
No difference in *B. pseudomallei* phagocytosis or killing capacity between WT and MyD88 KO cells. (A) Peripheral blood neutrophils were incubated at 37°C with CFSE-labeled growth-arrested *B. pseudomallei* (1×10^7^ CFU/ml) after which time-dependent phagocytosis was quantified; 1×10^4^ neutrophils were analysed per sample (see Methods section). (B) Killing capacity of peritoneal macrophages are shown as percentage of killed *B. pseudomallei* compared to t = 0. Data are mean±SEM; n = 5 per mouse strain. Open rounds represent WT cells, while black squares represent MyD88 KO mice; ns denotes not significant.

The experiments described above established the key role of MyD88 in the antibacterial defense towards *B. pseudomallei* infection. MyD88 has been described to play an important role in phagocytosis and killing of invading bacteria [26] and since *B. pseudomallei* is a facultative intracellular bacterium [13], [29]–[31] we next determined whether MyD88 contributes to phagocytosis and/or killing of *B. pseudomallei*. Interstingly, we found that MyD88 deficient neutrophils demonstrated an unaltered capacity to phagocytose *B. pseudomallei* (**Fig. 7A**). In addition, no difference in the killing capacity between WT and MyD88 deficient macrophages was observed (**Fig. 7B**).

### MyD88 plays an important role in the early neutrophil recruitment after infection with B. pseudomallei

**Figure 8:**
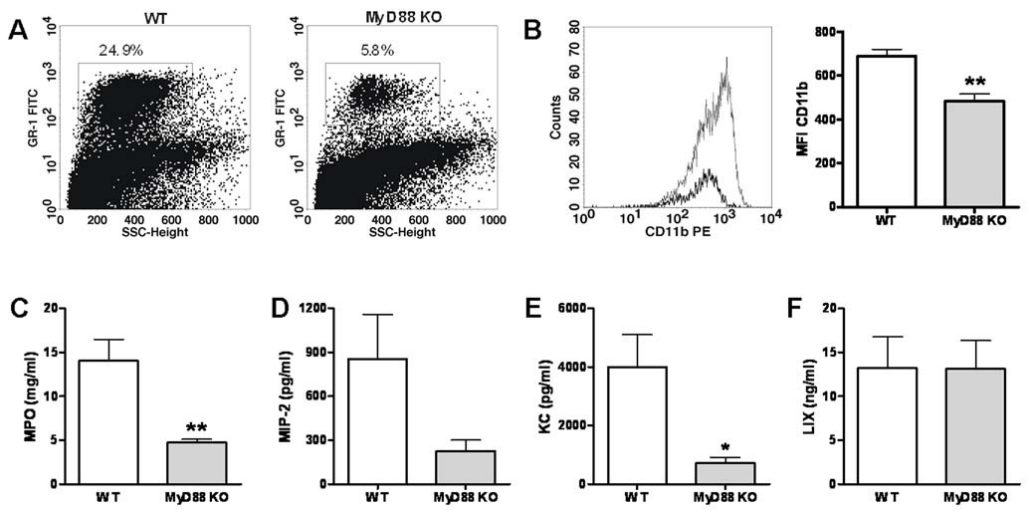
MyD88 plays an important role in early neutrophil recruitment after infection with *B. pseudomallei*. The impact of MyD88 deficiency on early neutrophil recruitment was investigated by analysing the amount of neutrophils in the pulmonary compartment using FACS analysis (see Methods) 24 h after intranasal inoculation of WT and MyD88 KO mice with 5×10^2^ CFU *B. pseudomallei*. MyD88 KO mice displayed significantly fewer neutrophils in their lungs compared with WT mice (the percentages of neutrophils of the total pulmonary cell count are given from one respresentative WT and one respresentative MyD KO mouse) (A). Additionally, MyD88 deficient neutrophils (gray line and gray bars) present at t = 24 expressed less CD11b on their surface compared to WT neutrophils (black line and white bars): representative histograms show decreased CD11b expression on pulmonary neutrophils (B). This corresponded with lower MPO levels in lung homogenates of MyD88 KO mice (gray bars) compared to controls (white bars) (C). In line, pulmonary MIP-2 (D) and KC (E) levels tended to be lower or were significantly reduced in MyD88 KO mice. LIX levels were unaltered in MyD88 KO mice at this early time point (F). SSC, side scatter; FITC: fluorescein isothiocyanate; PE, phycoerythrin; MFI, mean fluorescence intensity; MPO, Myeloperoxidase; MIP-2: macrophage-inflammatory protein-2; LIX, lipopolysaccharide-induced CXC chemokine. Bar figures represent mean±SEM; n = 6–8 per mouse strain. * *P*<0.05; ** *P*<0.01.

To further dissect the mechanism for the strong protective role of MyD88 during experimental melioidosis and in light of recent data suggesting that MyD88 is necessary for early neutrophil recruitment during hypersensitivity pneumonitis [32] as well as pulmonary infection with *Pseudomonas aeruginosa* [33], we investigated the role of MyD88 in neutrophil recruitment in our model. Therefore, we compared the pulmonary cell influx in WT and MyD88 KO mice after inoculation with *B. pseudomallei*. At 24 hours after infection, MyD88 KO mice had significantly fewer neutrophils in their lungs compared with WT mice (**Table 2** **and** **Fig. 8A**). In addition to a lower absolute amount of neutrophils in MyD88 KO mice, the neutrophils that were recruited were also less activated as demonstrated by both diminished CD11b positivity (**Fig. 8B**) and decreased pulmonary MPO content (**Fig. 8C**). In line, local levels of the neutrophil attracting chemokines MIP-2 and KC tended to be lower or were significantly reduced in MyD88 KO mice at 24 hours after infection (**Fig. 8D–E**). The pulmonary levels of LIX, a murine neutrophil-chemoattractant CXC chemokine [34], did not differ between WT and MyD88 KO mice at this early time point (**Fig. 8F**). Together these data suggest that the protective effect of MyD88 in the host defense against melioidosis can at least in part be explained by MyD88 dependent neutrophil recruitment following exposure to *B. pseudomallei*.

**Table 2:** Effect of MyD88 deficiency on total and differential lung cell counts.

	Leukocytes	Macrophages	Monocytes	Neutrophils	Lymphocytes/Other
	×10^5^/ml	%	%	%	%
**WT**	23±1	20±1	13±1	25±3	42±2
**MyD88 KO**	10±1	21±1	12±1	6±1**	60±1**

Total leukocyte counts (×10^5^/ml) and differential cell counts in lungs of wild-type (WT) and MyD88 knock-out mice 24 hours after intranasal infection with 5×10^2^ CFU of *B. pseudomallei*. Data are mean±SEM (n = 6–7/group); ** *P*<0.01 versus WT.

## Discussion

The identification of the TLR family in conjunction with their specific signaling pathways has led to an explosion of knowledge in understanding both the physiological and pathophysiological role of these innate immune signaling pathways in the event of sepsis [3], [4], [9]–[11]. Although our knowledge on the role of the innate immunity in the pathogenesis of sepsis caused by *B. pseudomallei* has progressed rapidly over the past years [13], much is unclear about which TLR signaling pathways are activated and how these TLR initiated signals are integrated into the more general infrastructure of the host defense in which they operate. The melioidosis mouse model in which *B. pseudomallei* is delivered per the intranasal route has been proven successful in mimicking pneumonia-derived septic melioidosis [13], [14], [17], [19]–[21], [35], [36] and the availability of MyD88 and TRIF deficient mice enabled us to further elucidate the predominant TLR signaling routes involved in melioidosis. We found that MyD88, but not TRIF, plays an important protective role in the host defense against *B. pseudomallei in vivo*. MyD88 deficient, but not TRIF mutant whole blood leukocytes released less TNFα upon stimulation with *B. pseudomallei* compared to WT cells. In addition, during experimental melioidosis MyD88 KO mice, but not TRIF mutant mice, demonstrated strongly increased lethality, accompanied by significantly increased bacterial loads when compared to WT mice. The decreased bacterial clearance capacity of MyD88 KO mice was accompanied by markedly reduced early pulmonary neutrophil recruitment and a diminished activation of neutrophils. These results further contribute to our understanding of the pathogenesis of sepsis in general and severe melioidosis in particular by revealing the important protective role of the MyD88 dependent signaling pathway by causing early neutrophil recruitment following *B. pseudomallei* exposure.

Our data are in line with other studies showing the crucial role of MyD88 in host defense against infection with both gram-positive [32], [37]–[39] and gram-negative bacteria [33], [40]–[44]. More specifically, our current results now further add to accumulating evidence that MyD88 is necessary for neutrophil recruitment during pneumonia [32], [33], [40], [42]. Neutrophils are known to play a critical role in the host defense against *B. pseudomallei* [36]. During murine melioidosis activated neutrophils are rapidly recruited to the lungs after intranasal infection with *B. pseudomallei* [17], [36]. In addition, depletion of neutrophils with anti-GR-1+ antibody severely exacerbated disease and was associated with a 1000-fold increase in pulmonary bacterial loads within 4 days [36]. We now show that during experimental melioidosis MyD88 is crucially involved in protective neutrophil recruitment. We further extend these findings by showing the importance of MyD88 in the activation of neutrophils as demonstrated by reduced CD11b expression on MyD88 KO neutrophils. The early diminished neutrophil recruitment was accompanied by reduced levels of the CXC chemokines KC and MIP-2, but unaltered early LIX levels, suggesting that MyD88 deficiency at least in part diminishes neutrophil influx during pneumonia derived melioidosis due to an attenuated production of CXC chemokines at the primary site of the infection. Notably, the observed decrease in neutrophil recruitment in the infected MyD88 KO mice was associated with decreased levels of TNFα, MCP-1 and IL-10 signifying that MyD88 is required for the induction of these cytokines. Of interest, the levels of some cytokines, most notably IL-6 and MCP-1, tended to be higher in MyD88 KO compared to WT mice at 72 hours after inoculation (non significant). Clearly, MyD88-independent pathways are also involved in proinflammatory cytokine production, which is in line with observations in MyD88 KO mice after pulmonary infection with *Haemophilus influenzae* [43].

Interestingly, we also show that MyD88 does not play a role in phagocytosis or killing of the facultative intracellular *B. pseudomallei*. This is in contrast with one earlier report describing MyD88 to be important in phagocytosis and killing of invading bacteria [26], but does correspond with more recent evidence arguing against a role of TLR-signalling in phagocytosis [45], [46]. One of the ways in which phagocytic cells are able to kill engulfed microbes is through the production of bactericidal reactive oxygen species via the NADPH oxidase enzyme complex [47], [48]. Breitbach *et al.* convincingly demonstrated that NADPH oxidase is essential in controlling intracellular *B. pseudomallei* [49]. Mice that are deficient for *gp91phox*, one of the two membrane bound components of the NADPH oxidase complex, have a accelerated mortality after infection with *B. pseudomallei* [49]. Given the fact that NADPH oxidase function is partially controlled by MyD88 [47], it is surprising that we were unable to detect a difference in intracellular numbers of *B. pseudomallei* between WT and MyD88 deficient macrophages after one hour of incubation. We cannot exclude that MyD88 dependent modulation of NADPH oxidase activity does play a role at later time point after cellular infection. Further research is needed to dissect the interaction between MyD88 and NADPH oxidase during melioidosis.

Remarkably, MyD88 KO and WT mice displayed similar lung pathology at 72 hours after infection. Thus, neutrophil recruitment was relatively selectively influenced by MyD88 deficiency in the context of the lung inflammatory response as a whole. It should be noted that MyD88 KO mice had much higher bacterial loads in their lungs than WT mice, and one could therefore argue that MyD88 KO mice had a relatively attenuated inflammatory pulmonary response. Nonetheless, our data indicate that even in the absence of MyD88 signaling, which abrogates the function of all relevant TLRs as well as of the IL-1R and IL-18R, *B. pseudomallei* is still able to elicit profound inflammation in the pulmonary compartment. Further research is warranted which MyD88 independent components of the innate immune system contribute herein.

Bacterial dissemination to the liver and the formation of liver abscesses is one of the hallmarks of melioidosis septic shock [12], [13]. We now show that MyD88 KO mice display a markedly increased susceptibility to liver injury as indicated by increased histopathology scores and clinical chemistry levels. The MyD88 pathway has recently been shown to be essential for early liver restoration after partial hepatectomy [50]. Furthermore, in a model of sepsis caused by polymicrobial infection both systemic and hepatic inflammatory responses were strongly attenuated in the absence of MyD88 [51]. Our current data indicate that in melioidosis MyD88 deficiency results in increased hepatocellular injury most likely due to the much higher bacterial loads in the liver. In addition, these data suggest that MyD88 dependent signaling is not required for the occurrence of liver inflammation and injury.

In addition of being the key signalling adaptor of virtual all TLRs, MyD88 also serves as the main adaptor molecule for the IL-1R and the IL-18R [5]. It has been shown *in vitro* that *B. pseudomallei* is capable of inducing caspase-1 dependent death in macrophages which is accompanied by the release of IL-1β and IL-18 [52]. Indeed, both IL-1β and IL-18 are known to be upregulated during melioidosis [20], [53], [54]. Our current data can now be brought in line with more recent findings showing the importance of IL-18 in the host defense against melioidosis: mice treated with anti-IL18R antibody [55] or IL-18 KO mice [20] have similar phenotypes as MyD88 KO mice as they all show a markedly increased susceptibility to infection with *B. pseudomallei*. It remains to be established to what extent TLR dependent MyD88 signaling contributes to the phenotype of MyD88 KO mice in experimental melioidosis. We have previously reported on the role of TLR2 and TLR4 in the host defense against *B. pseudomallei* and found that although both TLR2 and TLR4 contribute to cellular responsiveness to *B. pseudomallei in vitro* only TLR2 impacts on the immune response of the intact host *in vivo* [14]. Specifically, TLR2 KO mice were strongly protected against *B. pseudomallei* induced mortality, which was accompanied by much lower bacterial loads in lungs and livers [14]. Since TLR2 signaling fully relies on MyD88 [3], [5], these results imply that complete MyD88 deficiency overrules the protective effect of TLR2 restricted MyD88 signaling. Our own preliminary results do not point toward an important role for TLR9 in host defense against *B. pseudomallei* (Wiersinga W.J. et al, unpublished). A likely MyD88 dependent candidate within the TLR family as a mediator of protective immunity against melioidosis is TLR5, which recognizes flagellin, an important component of *B. pseudomallei* [13], [56]. The first studies using TLR5 KO mice are about to be initiated in our laboratory. Together, these data indicate that MyD88 deficiency results in a strongly impaired resistance against melioidosis likely due to a combined deficiency in IL-1R, IL-18R and possibly TLR5 signaling, and in spite of an interruption of harmful TLR2 signaling.

Two earlier studies documented a reduced innate immune response and a diminished bacterial clearance in TRIF deficient mice with experimentally induced gram-negative pneumonia caused by either *Escherichia coli* or *Pseudomonas aeruginosa* [57], [58]. Our laboratory, however, reported that MyD88 but not TRIF is important in clearing non-typeable *Haemophilus influenzae* from the mouse lung [43]. We now show that TRIF does not play a role in the host defense against *B. pseudomallei*, which is in full correspondence with the earlier found limited role of TLR4 in this gram-negative infection [14].

We conclude that MyD88 dependent signaling contributes to a protective host response against *B. pseudomallei* at least in part by causing early neutrophil activation and recruitment towards the site of infection. In addition our study reveals that the role of TRIF signaling is of minor importance for the clearance of infection with *B. pseudomallei*.
